# Use of integrative and complementary health practices by Brazilian population: results from the 2019 National Health Survey

**DOI:** 10.1186/s12889-023-16083-y

**Published:** 2023-06-15

**Authors:** Rodrigo Garcia-Cerde, Pollyanna Fausta Pimentel de Medeiros, Leonardo F. Silva, Juliana Y. Valente, Solange Andreoni, Zila M. Sanchez, Leandro F. M. Rezende

**Affiliations:** 1 Department of Preventive Medicine, Universidade Federal de São Paulo. Address: Rua Botucatu, 740 - 4° Andar, São Paulo, SP CEP: 04023-062 Brazil; 2 Institute of Health and Society, Universidade Federal de São Paulo. Address: Rua Silva Jardim 136, Santos, SP CEP: 11015-020 Brazil; 3 Department of Psychiatry, Universidade Federal de São Paulo. Address: Rua Borges Lagoa, 570 - 1º Andar, São Paulo, SP CEP: 04038-000 Brazil

**Keywords:** Integrative medicine, Alternative health practices, Comprehensive medicine, Complementary therapies, Cross-sectional study, Nationally representative data

## Abstract

**Background:**

In 2006, Brazil implemented the National Policy on Integrative and Complementary Practices of the SUS. and in 2015, the Brazilian Ministry of Health issued a reinforcement to this policy to increase access to integrative and complementary health practices (ICHP). In this study, we described the prevalence of ICHP in Brazilian adults according to their sociodemographic characteristics, self-perceived health, and chronic diseases.

**Methods:**

This is a nationally representative cross-sectional survey including 64,194 participants from the 2019 Brazilian National Health Survey. Types of ICHP were categorized according to their purposes: health promotion (Tai chi/Lian gong/Qi gong, yoga, meditation, and integrative community therapy) or therapeutic practices (acupuncture, auricular acupressure, herbal treatment and phytotherapy, and homeopathy). Participants were classified as non-practitioners and practitioners, who in turn were grouped according to use of ICHP in the last 12 months: only used health promotion practices (HPP); only used therapeutic practices (TP); used both (HPTP). Multinomial logistic regressions were performed to estimate the associations of ICHP with sociodemographic characteristics, self-perceived health status, and chronic diseases.

**Results:**

Brazilian adults showed an ICHP use prevalence of 6.13% [95%CI = 5.75–6.54]. Compared to non-practitioners, women and middle-aged adults were more likely to use any ICHP. Afro-Brazilians were less likely to use both HPP and HPTP, whereas Indigenous people were more likely to use both HPP and TP. We found a positive gradient of association among participants with higher income and educational attainment and access to any ICHP. People from rural areas and those with negative self-perceived health were more likely to use TP. Participants with arthritis/rheumatism, chronic back problems, and depression were more likely to use any ICHP.

**Conclusions:**

We found that 6% of Brazilian adults reported using ICHP in the previous 12 months. Women, middle-aged individuals, chronic patients, people with depression, and wealthier Brazilians are more likely to use any type of ICHP. Of note, rather than suggesting to expand the offer of these practices in the Brazilian public health system, this study diagnosed Brazilians’ behavior of seeking for complementary healthcare.

**Supplementary Information:**

The online version contains supplementary material available at 10.1186/s12889-023-16083-y.

## Background

The health-disease process is complex, and the traditional biomedical model has been sometimes insufficient to respond to the population’s health-care demands. In this context, healthcare providers and patients have used integrative and complementary health practices (ICHP) as a complement to health care [1]. ICHP are therapeutic resources that seek to prevent diseases and recover health by emphasizing an emphatic listening (thus developing the therapeutic bond) and integrating persons with their environment and society [2]. Since 2002, the World Health Organization (WHO) has encouraged its member countries to implement traditional medicine (TM), alternative and complementary medicine (ACM), and integrative medicine (IM) practices in primary health care [3]. Despite international recommendations, it is important to note the absence of evidence of the effectiveness of some practices included under the ICHP concept.

While Brazil uses the term ICHP, the WHO employs traditional, complementary, and integrative medicine (TCIM) [4]. In 2019, the WHO published its Global Report on TCIM, finding that 98 of its 194 member states had a national policy on this topic. Indigenous TM was the most common practice, followed by acupuncture, herbal medicine, chiropractic care, and homeopathy [5].

A systematic review conducted in 32 countries estimated an ICHP prevalence of 26.4% [6]. A study conducted with patients with chronic non-communicable diseases (NCDs) in a Turkish hospital found that 63% of hypertensive patients used ICHP to improve their health condition [7]. Despite receiving primary care for hypertension, a survey conducted in Malaysia observed a 30.6% prevalence of raw herbs use in patients with hypertension as a way to control it [8]. In Brazil, about 4 to 5% of its general adult population used ICHP in 2013 [4, 9].

The Brazilian Unified Health System (*Sistema Único de Saúde*—*SUS*)—a national, universal, public, and free health system—implemented the National Policy on Integrative and Complementary Practices (PNPIC) in 2006, based on experiences in Brazilian states and municipalities, proposals of several National Health Conferences, and WHO recommendations [10]. Since that year, Brazilian scientific publications addressing this topic have increased [11]. The main purpose of PNPIC is to promote and monitor the Brazilian population’s use of ICHP by SUS programmatic offers. In 2015, the Ministry of Health issued a reinforcement to this policy to increase access to ICHP at SUS [12]. In 2019, Brazil conducted its second National Health Survey (*Pesquisa Nacional de Saúde – PNS*), a large national representative household survey whose main objective was to provide information on the determinants, conditions, and health needs of the Brazilian population [13]. This study intends to add information on the use of ICHP, rather than discussing its effectiveness or its accessibility increase at SUS.

We aimed to describe the use of ICHP in the Brazilian population (≥ 15 years) according to their purpose (health promotion or therapeutic). We also described sociodemographic characteristics, self-perceived health, and chronic diseases associated with ICHP use.

## Methods

### Study design, population, and sample size

This is a cross-sectional descriptive study with national-representative data from the PNS, conducted in 2019 by the Brazilian Ministry of Health (BMH) along with the Brazilian Institute of Geography and Statistics (IBGE). The PNS used a complex sampling strategy in three selection stages: 8036 census tracts or sets of tracts (Primary Sampling Unit – PSU) were randomly selected based on the IBGE census, totaling 53% of all PSU. Private households were selected from a registry of national addresses by simple random sampling. Finally, one resident from each household aged ≥ 15 years was randomly selected to compose the set of units in the third stage. Details on the sampling and weighting process were made available in a previous publication [14]. The selected sample consisted of 108,457 households and 90,846 interviewed participants, with 91.9 and 95.6% overall response rates, respectively. This study uses the information of 64,290 participants (aged ≥ 15 years) who responded to a questionnaire on ICHP.

### Ethics

Interviewers were trained to ensure the confidentiality of the identity and personal data of household residents and interviewees. Informed consent was obtained in two stages: before collecting the information given by household informants (proxy) and when a household resident aged 15 years or above was selected for an individual interview and anthropometric measurements. The 2019 PNS project was submitted to the Brazilian National Research Ethics Committee/National Health Council and approved under opinion no. 3.529.376 (August 23^rd^, 2019).

### Integrative and complementary health practices

ICHP were measured by the following question: “In the last 12 months, did you use… [acupuncture, auricular acupressure, herbal treatment and phytotherapy, homeopathy, Tai Chi/Lian gong/Qi gong, yoga, meditation, or integrative community therapy?]” (*yes* or *not*). For analytical purposes, these practices were classified into two groups according to the ICHP purpose and as descripted in the BMH website [2]: the first group referred to “health promoting activities,” whose main objective was to preserve or increase practitioners’ health (Tai Chi/Lian gong/Qi gong, yoga, meditation, and integrative community therapy) [15], and the second group, to “therapies,” whose main purpose was to provide treatment for a disease or condition (acupuncture, auricular acupressure, herbal treatment and phytotherapy, and homeopathy). Participants were classified into four groups: (i) non-practitioners, (ii) used only health promotion practices (HPP); (iii) used only therapeutic practices (TP); and (iv) used both HPP and TP (HPTP).

### Sociodemographic characteristics

Information on sex (men and women), age (from 15 to 104 years: ≤ 24, 25 to 34, 35 to 44, 45 to 54, 55 to 64, 65 to 74, and ≥ 75), ethnicity/skin color (Caucasian/white, *Pardo*/brown, Afro Brazilian/black, Asian Brazilian/yellow, and Indigenous), per capita household income group (≤ 0.5 monthly minimum wage, 0.6 to 2 monthly minimum wages, 2.1 to 5 monthly minimum wages, and > 5.1 monthly minimum wages), educational attainment (none or incomplete primary education, complete primary or incomplete secondary education, complete secondary or incomplete undergraduate, and university graduate), geographical accessibility (urban and rural area), and health system accessibility (public or private) were collected in the questionnaire.

### Self-perceived health and chronic diseases

Self-perceived health status was measured via the question: “In general, how is the state of your health?” The options were very good, good, regular, bad, and very bad. For analytical purposes, this variable was categorized into three groups: good/very good, regular, and bad/very bad. To confirm a medical diagnosis of chronic diseases, was asked: “Has any doctor ever given you a diagnosis of… [diabetes, hypertension, high cholesterol, heart disease, cerebrovascular accident (CVA), asthma or asthmatic bronchitis, arthritis or rheumatism, chronic back problems, depression, lung disease, cancer, or chronic renal insufficiency?]”.

### Statistical analysis

Prevalence and 95% confidence intervals (CI) were used in the descriptive analyses. Sociodemographic characteristics, self-perceived health status, and chronic diseases were described according to ICHP groups. The prevalence of ICHP was also described according to sociodemographic characteristics, self-perceived health status, and chronic diseases.

Multinomial multivariable logistic regression models were performed to estimate odds ratios (OR) and their 95%CI regarding associations between sociodemographic characteristics, self-perceived health status, chronic diseases, and ICHP use. ICHP was the dependent variable. Moreover, a sensitivity analysis was performed by running multivariable multinomial logistic models using a hierarchical approach, as indicated by Victora et al. [16]. In the distal model, the included variables were accessibility, sex, age, ethnicity, per capita household income, and educational attainment; [2] in the intermediate model, the distal model with chronic diseases; and [3] in the proximal model, the distal and intermedial models and self-perceived health status.

All statistical analyses were performed using Stata 17 (StataCorp. 2021. Stata Statistical Software: Release 17. College Station, TX: Stata Corp LLC). The complex sampling design was considered in all analyses using the “svyset” command, which considers sample weights. Microsoft Excel (Microsoft Corporation. 2016. Excel 2016. Software) was used to create the figures.

## Results

We found a 6.13% (95%CI = 5.75–6.54) use prevalence of any Integrative and Complementary Health Practice (ICHP) in 2019. The TP group was the most prevalent (4.90%, 95%CI = 4.57–5.25). We found a higher proportion of women in all three ICHP groups than in non-practitioners. Participants using TP were older than their ICHP counterparts. We found a higher proportion of Caucasian (white) individuals in all three ICHP groups, especially HPP (68.2%) and HPTP groups (69.4%), whereas we found a higher proportion of *Pardo* (Brown) individuals in non-practitioners (44.9%). HPP and HPTP participants had higher monthly incomes (2.1 monthly wages or more) and educational attainment (University graduate: 60.7% and 63.8%, respectively) than non-practitioners (Table 1).

**Table 1:** Participants sociodemographic characteristics according to use of integrative and complementary practices. 2019 National Health Survey, Brazil

			Integrative and complementary practices					
Characteristics	Non-practitioners		Practitioners					
	*N* = 59,703 93.87% [93.47; 94.25]		*N* = 4,481 6.13% [5.75; 6.54]					
			Only health-promoting practices^a^		Only therapeutic practices^b^		Health-promoting & therapeutic practices	
			*N* = 342 0.57% [0.48; 0.68]		*N* = 3,667 4.90% [4.57; 5.25]		*N* = 472 0.66% [0.55; 0.79]	
	n	% [95%CI]	n	% [95%CI]	n	% [95%CI]	n	% [95%CI]
*Sex*								
Men	25,266	47.7 [47.5; 47.9]	91	31.0 [23.3; 40.0]	1,283	37.2 [34.1; 40.3]	109	28.3 [20.4; 38.0]
Women	34,437	52.3 [52.1; 52.5]	251	69.0 [60.1; 76.8]	2,384	62.8 [59.7; 65.9]	363	71.7 [62.1; 79.7]
*Age*	mea*N* = 43.2 [43.1; 43.3]		mea*N* = 40.7 [38.1; 43.4]		mea*N* = 47.4 [46.1; 48.6]		mea*N* = 43.8 [41.2; 46.5]	
≤ 24	4,737	18.8 [18.6; 19.0]	31	20.9 [13.7; 30.5]	189	12.9 [10.0; 16.4]	24	10.8 [6.2; 18.2]
25 to 34	10,369	17.1 [16.7; 17.4]	81	20.7 [14.8; 28.1]	476	12.2 [10.5; 14.1]	96	23.3 [15.9; 32.9]
35 to 44	11,752	19.1 [18.6; 20.0]	83	21.0 [15.5; 27.9]	703	20.2 [17.7; 22.9]	110	20.0 [15.2; 25.8]
45 to 54	10,761	16.9 [16.5; 17.2]	65	18.0 [12.3; 25.7]	703	19.6 [17.2; 22.4]	80	13.6 [9.6; 18.9]
55 to 64	10,490	14.5 [14.1; 14.9]	41	9.4 [6.2; 14.1]	751	15.7 [14.1; 17.6]	96	23.7 [16.5; 32.7]
65 to 74	7,517	9.1 [8.8; 9.4]	27	5.5 [3.3; 9.0]	580	13.1 [11.5; 14.9]	47	6.3 [4.0; 9.6]
≥ 75	4,077	4.7 [4.5; 4.9]	14	4.5 [2.2; 8.9]	265	6.3 [5.0; 8.0]	19	2.4 [1.2; 4.8]
*Ethnicity (raciality)*								
Caucasian (white)	20,998	40.1 [39.3; 40.9]	217	68.2 [60.1; 75.4]	1,319	46.7 [43.2; 50.3]	296	69.4 [61.7; 76.1]
Pardo (brown)	30,743	44.9 [45.2;46.8]	92	23.1 [16.7;31]	1,881	40.7 [37.4; 43.9]	133	21.9 [16.5; 28.6]
Afro Brazilian (black)	7,027	12.4 [11.8; 12.9]	23	4.3 [2.5; 7.4]	387	10.7 [9.1; 12.4]	33	5.6 [3.3; 9.2]
Asian Brazilian (yellow)	457	1.1 [0.9; 1.3]	7	2.9 [1.2; 7.2]	31	0.9 [0.5; 1.4]	8	3 [1; 8.4]
Indigenous	469	0.5 [0.4; 0.6]	3	1.3 [0.4; 4.7]	49	1.1 [0.6; 1.9]	2	0.1 [0.02; 0.4]
*Per capita household income* ^c^								
≤ 0.5 monthly wage	15,378	23.9 [23.3; 24.5]	17	4.4 [2.1; 8.8]	844	18.6 [16.4; 21.1]	26	3.3 [1.5; 7.2]
0.6 to 2 monthly wages	32,412	56.5 [55.7; 57.3]	85	27.7 [20.6; 36.1]	1,754	49.3 [45.8; 52.8]	130	30.9 [22.8; 40.4]
2.1 to 5 monthly wages	8,656	14.8 [14.2; 15.4]	107	30.6 [23.7; 38.6]	673	21.9 [19.2; 24.9]	167	40.8 [32.3; 49.9]
> 5.1 monthly wages	3,248	4.8 [4.4; 5.2]	132	37.3 [29.1; 46.3]	396	10.1 [8.4; 12.2]	149	25 [19.4; 31.6]
*Educational attainment*								
None or incomplete primary education	25,009	34.8 [34.1; 35.6]	23	4.8 [2.8; 8]	1,483	31.7 [28.7; 34.7]	28	6.1 [3.4; 10.8]
Complete primary or incomplete secondary education	8,493	17.7 [17.1; 18.2]	9	5.8 [2.4; 13]	378	11.4 [9.7; 13.3]	16	5.7 [2.6; 12.1]
Complete secondary or incomplete undergraduate course	17,569	33.2 [32.5; 33.9]	88	28.8 [21.6; 37.2]	956	32.6 [29.2; 36.1]	106	24.4 [16.9; 33.8]
University graduate	8,632	14.3 [13.6; 15]	222	60.7 [52.2; 68.5]	850	24.4 [21.7; 27.3]	322	63.8 [54.4; 72.3]
*Accessibility*								
Urban area	45,589	85.0 [84.5; 85.4]	328	97.4 [94.7; 98.7]	2,652	83.8 [81.5; 85.9]	457	97.4 [94.0; 98.9]
Rural area	14,114	15.0 [14.6; 15.5]	14	2.6 [1.3; 5.3]	1,015	16.2 [14.1; 18.5]	15	2.6 [1.1; 6.0]
Received in the SUS^d^	-	-	29	9.0 [5.4; 14.6]	202	6.0 [4.9; 7.4]	31	5.4 [2.9; 10.1]
Received privately	-	-	312	91.0 [85.4; 94.6]	3,461	93.97 [92.6; 95.1]	440	94.6 [90.0; 97.1]

*Abbreviations*: “N” or “n”: sample size; “95% CI”: 95% confidence intervals

^a^Include: Tai chi chuan/Lian gong/Qi gong (*n* = 62), yoga (*n* = 369), meditation (*n* = 607), and integrative community therapy (*n* = 85)

^b^Include: Acupuncture (*n* = 1,012), auricular acupressure (*n* = 239), herbal treatment and phytotherapy (*n* = 3,303), and homeopathy (*n* = 651)

^c^Minimum monthly salary in Brazilian currency in 2019: R$ 998,00 [https://legislacao.presidencia.gov.br/atos/?tipo=DEC&numero=9661&ano=2019&ato=472ETWq5keZpWT17b]

^d^“SUS”: Brazilian Unified Public Health Care System

Participants in the HPP (85.6%) and HPTP groups (81.5%) were more likely to report good/very good self-perceived health than non-practitioner (68.3%). We observed a higher prevalence for all chronic diseases in the TP group (Table 2).

**Table 2:** Participants self-perceived health and chronic diseases status according to integrative and complementary practices. 2019 National Health Survey, Brazil

			Integrative and complementary practices					
Characteristics	Non-practitioners		Practitioners					
	*N* = 59,703 93.87% [93.47; 94.25]		*N* = 4,481 6.13% [5.75; 6.54]					
			Only health-promoting practices^a^		Only therapeutic practices^b^		Health-promoting & therapeutic practices	
			*N* = 342 0.57% [0.48; 0.68]		*N* = 3,667 4.90% [4.57; 5.25]		*N* = 472 0.66% [0.55; 0.79]	
	n	% [95%CI]	n	% [95%CI]	n	% [95%CI]	n	% [95%CI]
*Self-perceived health status*								
Good / very good	36,670	68.3 [67.6; 69]	289	85.6 [79.4; 90.1]	1,861	56.9 [53.8; 60]	377	81.5 [74.6; 86.8]
Regular	18,792	26.4 [25.7; 27]	44	10.9 [7.2; 16.2]	1,388	33.8 [31.1; 36.7]	82	16.4 [11.3; 23.3]
Bad / very bad	4,241	5.3 [5; 5.6]	9	3.5 [1.4; 8.4]	418	9.3 [7.5; 11.5]	13	2.1 [0.9; 4.9]
*Chronic diseases*								
Diabetes	5,276	8.0 [7.7; 8.4]	20	4.8 [2.5; 9.0]	375	9.7 [7.7; 12.2]	25	2.9 [1.6; 5.1]
Hypertension	16,927	24.3 [23.8; 24.8]	65	17.7 [12.3; 24.7]	1,285	33.2 [30.3; 36.3]	107	20.3 [14.7; 27.3]
High cholesterol	9,373	14.6 [14.0; 15.1]	60	15.0 [10.4; 21.0]	852	23.4 [20.4; 26.6]	92	17.5 [10.7; 27.2]
Heart disease	3,321	5.0 [4.7; 5.3]	15	4.1 [2.1; 7.8]	268	6.7 [5.5; 8.3]	36	7.2 [4.2; 12.1]
Cerebrovascular accident (CVA)	1,271	1.7 [1.5; 1.8]	6	1.5 [0.6; 3.9]	105	2.7 [1.8; 4.0]	10	1.6 [0.5; 4.6]
Asthma or asthmatic bronchitis	2,847	5.4 [5.1; 5.7]	35	7.6 [4.5; 12.6]	258	8.3 [6.3; 10.9]	62	9.8 [6.7; 14.2]
Arthritis or rheumatism	5,109	7.0 [6.6; 7.5]	27	6.2 [3.5; 10.6]	622	19.2 [16.3; 22.6]	60	12.8 [8.1; 19.6]
Chronic back problems	12,783	20.0 [19.4; 20.7]	72	22.1 [16.1; 29.6]	1,416	38.9 [35.6; 42.3]	138	32.0 [25.0; 39.9]
Depression	5,618	9.4 [9.0; 9.8]	77	29.1 [21.4; 38.3]	599	17.6 [15.2; 20.4]	135	29.6 [21.9; 38.7]
Lung disease	812	1.5 [1.4; 1.7]	10	2.1 [1.0; 4.4]	98	3.1 [1.7; 5.4]	11	2.3 [1.0; 5.1]
Cancer	1,564	2.2 [2.0; 2.4]	13	2.7 [1.1; 6.1]	160	4.2 [3.2; 5.4]	30	6.5 [2.6; 15.6]
Chronic renal insufficiency	868	1.4 [1.3; 1.6]	1	0.1 [0.0; 0.9]	91	2.2 [1.5; 3.3]	6	0.5 [0.2; 1.3]

*Abbreviations*: “N” or “n”: sample size; “95% CI”: 95% confidence intervals

^a^Include: Tai chi chuan/Lian gong/Qi gong (*n* = 62), yoga (*n* = 369), meditation (*n* = 607), and integrative community therapy (*n* = 85)

^b^Include: Acupuncture (*n* = 1,012), auricular acupressure (*n* = 239), herbal treatment and phytotherapy (*n* = 3,303), and homeopathy (*n* = 651)

**Fig. 1:**
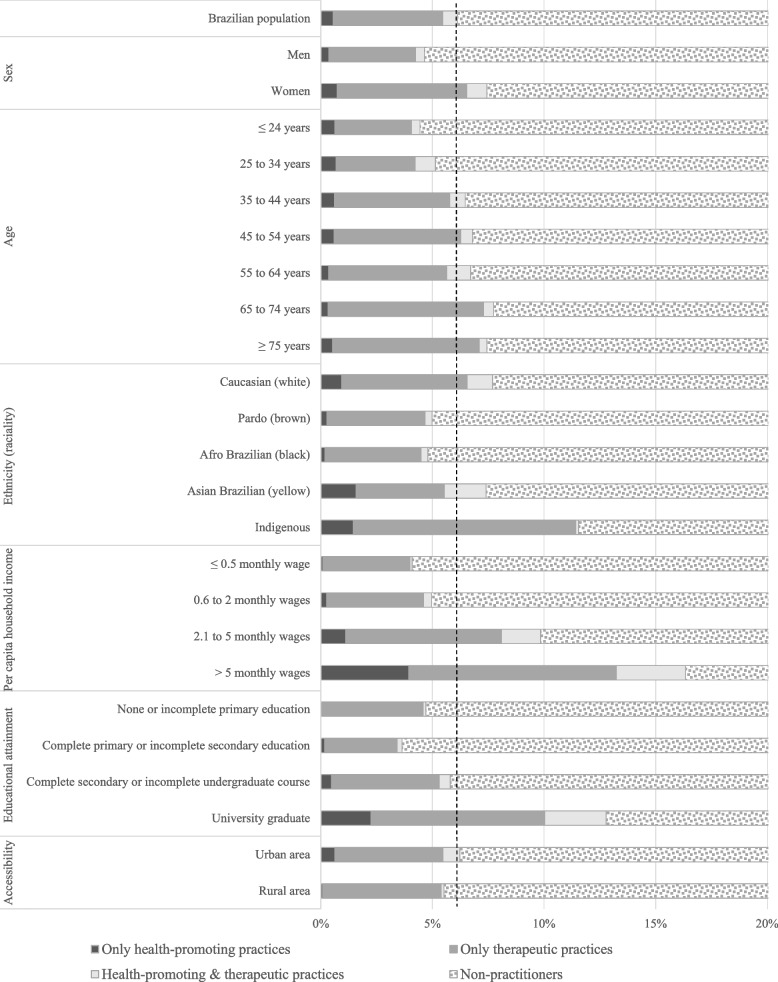
Prevalence of use of integrative and complementary practices by sociodemographic characteristics of the Brazilian population. 2019 National Health Survey, Brazil

**Fig. 2:**
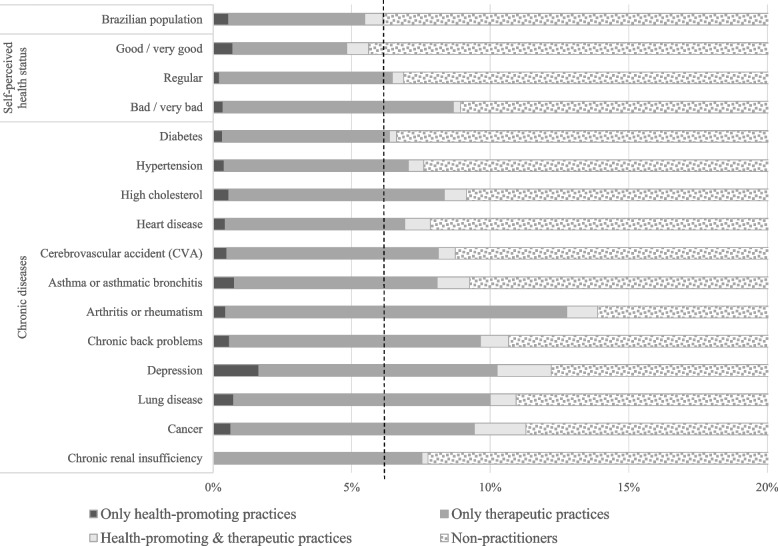
Prevalence of use of integrative and complementary practices by self-perceived health status and medical diagnosis of chronic diseases of the Brazilian population. 2019 National Health Survey, Brazil

Figures 1 and 2 show the prevalence of ICHP according to sociodemographic characteristics, self-perceived health status, and chronic diseases. We found a greater HPP prevalence in women; Asian individuals; those with higher incomes and educational attainment; inhabitants of urban areas; participants with good or very good self-perceived health; and those who received a diagnosis of depression. TP were more prevalent in women; older individuals (≥ 65 years); Indigenous people; those with higher incomes and educational attainment; inhabitants of rural areas; participants with poor or very poor self-perceived health; and those who received a diagnosis of arthritis or rheumatism diagnosis. HPTP had similar characteristics to HPP but included diagnosis of depression and cancer.

Table 3 shows the adjusted multivariable multinomial logistic regression using the non-practitioner group as reference group. Women were more likely to use HPP (OR = 2.31, 95%CI = 1.51–3.54) than men. At each increase of one year of life, the chance of belonging to the HPP and HPTP groups decreases by 2%. Afro-Brazilians had lower odds of using both HPP (OR = 0.45, 95%CI = 0.24–0.85) and HPTP (OR = 0.54, 95%CI = 0.30–0.96) than Caucasian/white individuals. Indigenous people had higher odds of using HPP (OR = 4.99, 95%CI = 1.51–16.54) and TP (OR = 2.60, 95%CI = 1.33–5.10) than Caucasian/white ones. We observed a positive gradient of association between per capita household income, educational attainment, and use of any ICHP group with a higher magnitude of association for HPP and HPTP. Participants living in rural areas had 48% higher odds of using TP (OR = 1.48, 95%CI = 1.22–1.79) than their urban counterparts. We observed an inverse dose–response association between self-perceived health status and TP. Finally, participants living with depression had higher odds of using HPP; participants diagnosed with cholesterol, arthritis or rheumatism, chronic back problems, and depression had higher odds of using TP; and patients with chronic back problems and depression had higher odds of using HPTP. On the other hand, chronic renal insufficiency was inversely associated with HPP and HPTP groups, heart disease with TP, and diabetes with HPTP group. Table S1 shows sensitive analysis with similar results.

**Table 3:** Adjusted multivariable multinomial logistic regression for the association of sociodemographic characteristics, self-perceived health status, and chronic diseases with use of integrative and complementary practices of Brazilian population

Characteristics		Only health-promoting practices *versus* Non-practitioners			Only therapeutic practices *versus* Non-practitioners			Health-promoting and therapeutic practices *versus* Non-practitioners		
	Overall *p*-value^a^	OR	95%CI	*p*-value	OR	95%CI	*p*-value	OR	95%CI	*p*-value
*Sex*	< 0.001									
Men		1			1			1		
Women		2.31	[1.51; 3.54]	< 0.001	1.30	[1.11; 1.52]	0.001	1.90	[1.21; 2.99]	0.005
Age	0.013	0.98	[0.96; 0.99]	0.002	1.00	[0.99; 1.01]	0.970	0.98	[0.97; 1.00]	0.024
*Ethnicity (raciality)*	0.327									
Caucasian (white)		1			1			1		
Pardo (brown)		0.77	[0.48; 1.23]	0.275	0.99	[0.83; 1.17]	0.883	0.71	[0.48; 1.04]	0.080
Afro Brazilian (black)		0.45	[0.24; 0.85]	0.014	0.91	[0.73; 1.14]	0.433	0.54	[0.30; 0.96]	0.037
Asian Brazilian (yellow)		1.68	[0.59; 4.74]	0.329	0.83	[0.47; 1.48]	0.535	2.06	[0.64; 6.65]	0.228
Indigenous		4.99	[1.51; 16.54]	0.009	2.60	[1.33; 5.10]	0.005	0.28	[0.07; 1.12]	0.072
*Per capita household income*	< 0.001									
≤ 0.5 monthly wage		1			1			1		
0.6 to 2 monthly wages		2.32	[0.96; 5.58]	0.060	1.06	[0.87; 1.29]	0.548	3.29	[1.18; 9.12]	0.022
2.1 to 5 monthly wages		5.41	[2.12; 13.80]	< 0.001	1.64	[1.27; 2.10]	< 0.001	7.83	[2.44; 25.13]	0.001
> 5.1 monthly wages		17.46	[6.22; 49.00]	< 0.001	2.05	[1.49; 2.82]	< 0.001	9.48	[2.75; 32.60]	< 0.001
*Educational attainment*	< 0.001									
None or incomplete primary education		1			1			1		
Complete primary or incomplete secondary education		1.00	[0.27; 3.65]	0.995	1.01	[0.81; 1.25]	0.963	2.67	[1.08; 6.55]	0.033
Complete secondary or incomplete undergraduate course		3.61	[1.87; 6.97]	< 0.001	1.59	[1.33; 1.91]	< 0.001	2.45	[1.18; 5.09]	0.016
University graduate		7.15	[3.23; 15.80]	< 0.001	2.13	[1.68; 2.70]	< 0.001	7.74	[3.21; 18.66]	< 0.001
*Accessibility*	< 0.001									
Urban area		1			1			1		
Rural area		0.66	[0.30; 1.46]	0.305	1.48	[1.22; 1.79]	< 0.001	0.52	[0.20; 1.34]	0.175
*Self-perceived health status*	< 0.001									
Good / very good		1			1			1		
Regular		0.62	[0.36; 1.07]	0.086	1.35	[1.17; 1.56]	< 0.001	0.81	[0.50; 1.34]	0.415
Bad / very bad		1.02	[0.30; 3.51]	0.974	1.52	[1.17; 1.98]	0.002	0.49	[0.21; 1.14]	0.097
*Chronic diseases*										
Diabetes	0.086	1.03	[0.51; 2.06]	0.944	0.90	[0.70; 1.16]	0.420	0.43	[0.23; 0.82]	0.011
Hypertension	0.386	1.12	[0.71; 1.78]	0.622	1.15	[0.98; 1.35]	0.092	0.96	[0.61; 1.52]	0.867
High cholesterol	0.114	0.94	[0.62; 1.44]	0.787	1.20	[1.01; 1.44]	0.039	0.94	[0.48; 1.82]	0.855
Heart disease	0.120	0.84	[0.38; 1.87]	0.667	0.77	[0.59; 0.99]	0.045	1.43	[0.75; 2.73]	0.279
Cerebrovascular accident (CVA)	0.279	2.46	[0.81; 7.48]	0.112	1.26	[0.79; 2.00]	0.327	1.68	[0.60; 4.70]	0.324
Asthma or asthmatic bronchitis	0.266	0.95	[0.50; 1.80]	0.882	1.28	[0.96; 1.72]	0.098	1.32	[0.85; 2.05]	0.210
Arthritis or rheumatism	< 0.001	0.85	[0.45; 1.59]	0.609	2.00	[1.46; 2.74]	< 0.001	1.73	[0.98; 3.05]	0.060
Chronic back problems	< 0.001	1.30	[0.78; 2.16]	0.319	1.89	[1.60; 2.23]	< 0.001	1.74	[1.18; 2.58]	0.005
Depression	< 0.001	3.09	[1.80; 5.29]	< 0.001	1.26	[1.03; 1.54]	0.022	2.75	[1.67; 4.53]	< 0.001
Lung disease	0.631	1.70	[0.67; 4.31]	0.267	1.20	[0.70; 2.07]	0.511	1.10	[0.42; 2.89]	0.840
Cancer	0.250	0.94	[0.36; 2.47]	0.897	1.25	[0.93; 1.68]	0.147	2.29	[0.81; 6.51]	0.120
Chronic renal insufficiency	0.058	0.13	[0.02; 0.97]	0.046	0.92	[0.57; 1.47]	0.718	0.34	[0.12; 0.99]	0.047

*Abbreviations*: “N” or “n”: sample size; “OR”: odds ratio; “95% CI”: 95% confidence intervals

^a^Adjusted Wald test for joint significance testing

## Discussion

In this study, we estimated the prevalence of ICHP in the Brazilian population and described the sociodemographic and health-related characteristics associated with different types of ICHP. We found a 6.13% prevalence of ICHP (in the previous 12 months) in the Brazilian population. Compared to non-practitioners, women and those who reported high income and educational attainment were associated with higher odds of using ICHP. People with a medical diagnosis of arthritis or rheumatism, chronic back problems, and depression were associated with higher odds of using any ICHP. Middle-aged participants were more likely to use HPP and any HPTP. Afro-Brazilians were less likely to use HPP and HPTP, whereas Indigenous individuals were more likely to use both HPP and TP. Participants from rural areas and with negative self-perceived health were more likely to use TP. The primary intention of this article was to provide a general overview of the use of these practices in the Brazilian population. However, it is necessary to consider that the different analyzed ICHP have varying proportions of usage and are also employed for different purposes, which may fail to align with our classification as health promotion practices and therapeutic practices. Subsequent studies can focus on analyzing each practice or group of practices with similar diagnostic and therapeutic methods.

Results from a previous study using data from the 2013 PNS [9] showed that 4% of Brazilian adults used ICHP in the 12 months prior to it. Our results suggest that the prevalence of use of ICHP in the Brazilian population (6%) increased in recent years. They remain lower than the international average (26.4%), reported in a systematic review conducted with information from 32 countries [6]. This increasing use of ICHP may be partially due to the efforts of the Brazilian Ministry of Health to increase access to these practices at SUS by its national policy [12]. However, the acceptance of the Brazilian population is increasing in private services, which has the greatest demand. Organizational challenges persist at SUS, such as the creation of a specific regulation to implement 29 ICHP so far recognized in the national policy in 2021 [2], as well as the institutional strengthening of national management [17]. However, there are inquiries about the cost-effectiveness of ICHP, especially its expansion and offering by SUS. Additionally, the health system has no systematic process for evaluating the effectiveness of ICHP, making such process more difficult, considering that various practices that have different levels of effectiveness evidence are included in the ICHP concept [18].

The higher use of ICHP by women and middle-aged people are consistent with previous studies in Brazil [9] and other countries [6, 19]. Possible explanations are related to middle-aged women being more likely to be caregivers for both their children and their parents, using ICHP to complement the health care of their family and their own [20]. Similarly, women have traditionally been assigned the social role of caregivers [21], and show better attitudes toward their own health care, seeking health care services more frequently than men [22].

Our findings regarding the low access of Afro-Brazilians to ICHP resemble a study in the Brazilian State of Minas Gerais [23], although their study used no national data. These findings could be explained by lack of information on the availability of healthcare services and the low income of Afro-Brazilian population. The higher prevalence of Indigenous people in the TP group may be due to their cultural relationship with herbal treatments, as showed by Moebus [24].

Our findings showed that participants with higher income and educational attainment had higher odds of using ICHP, particularly HPP. These findings agree with previous studies showing that ICHP women users are more likely to have higher educational attainment and annual income than female non-users [19]. Nevertheless, another study conducted in 32 countries found that lower socioeconomical and educational status were associated with a higher use of ICHP [6]. An explanation for these contradictory findings may be that the set of ICHP includes very different practices, including those taught by specialized professionals (which require expensive supplies) and others from popular culture, such as the use of medicinal plants.

Our study also found that people living in rural areas are likely to use TP. Similarly, a systematic review [19] found that using manual therapies were more common in rural populations. Additionally, access to conventional healthcare may also explain differences between rural and urban areas regarding the use of ICHP [25]. We observed that negative self-perceived health and diagnosis of arthritis or rheumatism, chronic back problems, and depression were associated with higher use of ICHP. Previous studies conducted in Brazil [9] and other countries [6] have reported similar findings.

The use of ICHP as an additional tool in primary health care is increasingly common and recognized by health systems as a cost-effective practice that contributes to prevent different health conditions in the USA, South Korea, and many European countries [26]. Similarly, a study conducted in the US found that a systemic change in the health care model and in the training process will be necessary to emphasize prevention and health promotion based on the use of ICHP in the treatment of different chronic diseases [27, 28].

The strengths of our study include our use of data from a large, representative population-based household survey, which allowed us to obtain the Brazilian population’s ICHP use prevalence and our association of sociodemographic and health-related characteristics associated with the use of different ICHP groups. Additionally, these findings may be helpful to monitor the use of ICHP in Brazil, evaluate its cost-effectiveness, and implement specific strategies to expand access to ICHP.

The limitations of this study are the following: the data we collected relied solely on self-reported measures, which could introduce potential biases, mainly related to misclassification of ICHP. Furthermore, response bias may have occurred, as individuals may have provided non-genuine responses due to “social desirability”, leading us to underestimate the occurrence of certain ICHP or overestimate others. The cross-sectional design of our study prohibits the establishment of causal relationships between variables. Additionally, this study is a secondary analysis conducted in relation to the primary objectives of the PNS. The PNS ignored institutionalized populations (e.g., hospitals and nursing homes). Lastly, the lack of long-term follow-up hinders our ability to examine temporal changes and draw definitive conclusions about the observed associations. Despite these limitations, our study provides valuable insights into the topic and lays a foundation for future research in this area.

## Conclusions

In conclusion, we found that approximately 6% of the Brazilian population used ICHP in the 12 months prior to this study. We identified sociodemographic and health-related characteristics associated with the use of different ICHP groups. Our results may have important implications for identifying aspects regarding access, effectiveness, and costs to implement ICHP at SUS. Of note, this study, rather than suggesting the expansion of the offer of these practices in the Brazilian National Health System, has diagnosed Brazilians’ health behavior.

## Supplementary Information

**Additional file 1.**

## Data Availability

The analyzed dataset in this study are available at the following web link: https://www.ibge.gov.br/estatisticas/sociais/saude/9160-pesquisa-nacional-de-saude.html?=&t=microdados.

## Abbreviations

ICHP: Integrative and complementary health practices
HPP: Health promotion practices
TP: Therapeutic practices
HPTP: Health promotion and therapeutic practices
WHO: World Health Organization
TM: Traditional medicine
ACM: Alternative and complementary medicine
IM: Integrative medicine
TCIM: Traditional, complementary, and integrative medicine
NCDs: Non-communicable diseases
*Sistema Único de Saúde** – **SU**S*: Unified Health System
PNPIC: National Policy on Integrative and Complementary Practices
*Pesquisa Nacional de Saúde** – **PN**S*: National Health Survey
*IBGE*: Brazilian Institute of Geography and Statistics
PSU: Primary Sampling Unit
CVA: Cerebrovascular accident
CI: Confidence intervals
OR: Odds ratios
