# Supplementary material for: Use of integrative and complementary health practices by Brazilian population: results from the 2019 National Health Survey
Source: BMC Public Health. 2023 Jun 15;23:1153. doi: 10.1186/s12889-023-16083-y (PMC10268350; doi:10.1186/s12889-023-16083-y)
Supplement: Supplementary file 1 — Additional file 1. [file 12889_2023_16083_MOESM1_ESM.docx]

SUPPLEMENTARY MATERIAL

Table S1. Sensitivity analysis. Multinomial logistic regression outcomes with hierarchical approach, for the association of sociodemographic characteristics, self-perceived health status, and chronic diseases with use of integrative and complementary practices.

|  |  |  |  |  |  |  |  |  |  |
| --- | --- | --- | --- | --- | --- | --- | --- | --- | --- |
|  | Only health-promoting practices  *versus*  Non-practitioners | | | Only therapeutic practices  *versus*  Non-practitioners | | | Health-promoting &  therapeutic practices  *versus*  Non-practitioners | | |
|  | OR | 95%CI | *p*-value | OR | 95%CI | *p*-value | OR | 95%CI | *p*-value |
| DISTAL MODEL (*n* = 64,165) ^a^ |  |  |  |  |  |  |  |  |  |
| *Accessibility* |  |  |  |  |  |  |  |  |  |
| Urban area | 1 |  |  | 1 |  |  | 1 |  |  |
| Rural area | 0.55 | [0.25; 1.20] | 0.135 | 1.47 | [1.22; 1.77] | <0.001 | 0.55 | [0.22; 1.38] | 0.202 |
| *Sex* |  |  |  |  |  |  |  |  |  |
| Men | 1 |  |  | 1 |  |  | 1 |  |  |
| Women | 2.60 | [1.77; 3.81] | <0.001 | 1.61 | [1.39; 1.87] | <0.001 | 2.69 | [1.76; 4.10] | <0.001 |
| *Age* | 0.98 | [0.97; 0.99] | 0.001 | 1.01 | [1.01; 1.02] | <0.001 | 0.99 | [0.98; 1.01] | 0.332 |
| *Ethnicity (raciality)* |  |  |  |  |  |  |  |  |  |
| Caucasian (white) | 1 |  |  | 1 |  |  | 1 |  |  |
| Pardo (brown) | 0.62 | [0.41; 0.96] | 0.030 | 0.95 | [0.81; 1.12] | 0.538 | 0.57 | [0.39; 0.83] | 0.004 |
| Afro Brazilian (black) | 0.40 | [0.22; 0.74] | 0.004 | 0.93 | [0.76; 1.14] | 0.514 | 0.50 | [0.28; 0.90] | 0.020 |
| Asian Brazilian (yellow) | 1.50 | [0.57; 3.98] | 0.410 | 0.72 | [0.41; 1.25] | 0.241 | 1.59 | [0.50; 5.03] | 0.428 |
| Indigenous | 3.80 | [1.05; 13.73] | 0.041 | 2.33 | [1.27; 4.30] | 0.006 | 0.26 | [0.06; 1.07] | 0.062 |
| *Per capita household income* |  |  |  |  |  |  |  |  |  |
| ≤ 0.5 monthly wage | 1 |  |  | 1 |  |  | 1 |  |  |
| 0.6 to 2 monthly wages | 2.06 | [0.93; 4.56] | 0.076 | 1.04 | [0.87; 1.25] | 0.673 | 2.60 | [1.05; 6.45] | 0.039 |
| 2.1 to 5 monthly wages | 5.60 | [2.31; 13.54] | <0.001 | 1.56 | [1.21; 2.01] | 0.001 | 6.78 | [2.28; 20.18] | 0.001 |
| > 5.1 monthly wages | 16.38 | [6.34; 42.34] | <0.001 | 1.93 | [1.38; 2.69] | <0.001 | 8.53 | [2.71; 26.89] | <0.001 |
| *Educational attainment* |  |  |  |  |  |  |  |  |  |
| None or incomplete primary  education | 1 |  |  | 1 |  |  | 1 |  |  |
| Complete primary or incomplete  secondary education | 1.49 | [0.55; 4.01] | 0.428 | 0.89 | [0.72; 1.10] | 0.283 | 1.52 | [0.56; 4.09] | 0.410 |
| Complete secondary or incomplete  undergraduate course | 3.04 | [1.66; 5.56] | <0.001 | 1.30 | [1.08; 1.56] | 0.005 | 2.62 | [1.24; 5.54] | 0.011 |
| University graduate | 6.20 | [3.02; 12.72] | <0.001 | 1.69 | [1.31; 2.17] | <0.001 | 8.20 | [3.34; 20.13] | <0.001 |
|  |  |  |  |  |  |  |  |  |  |
| INTERMEDIATE MODEL (*n* = 58,157) ^b^ |  |  |  |  |  |  |  |  |  |
| *Chronic diseases* |  |  |  |  |  |  |  |  |  |
| Diabetes | 0.97 | [0.49; 1.90] | 0.920 | 0.95 | [0.74; 1.22] | 0.699 | 0.41 | [0.22; 0.78] | 0.007 |
| Hypertension | 1.09 | [0.68; 1.74] | 0.720 | 1.18 | [1.01; 1.39] | 0.042 | 0.95 | [0.60; 1.48] | 0.809 |
| High cholesterol | 0.93 | [0.61; 1.42] | 0.737 | 1.22 | [1.02; 1.46] | 0.027 | 0.94 | [0.49; 1.81] | 0.853 |
| Heart disease | 0.81 | [0.37; 1.78] | 0.593 | 0.80 | [0.61; 1.03] | 0.080 | 1.37 | [0.72; 2.61] | 0.334 |
| Cerebrovascular accident (CVA) | 2.35 | [0.79; 7.03] | 0.125 | 1.32 | [0.83; 2.09] | 0.237 | 1.54 | [0.53; 4.52] | 0.429 |
| Asthma or asthmatic bronchitis | 0.94 | [0.50; 1.79] | 0.860 | 1.31 | [0.97; 1.76] | 0.078 | 1.31 | [0.85; 2.03] | 0.225 |
| Arthritis or rheumatism | 0.83 | [0.45; 1.54] | 0.557 | 2.10 | [1.55; 2.85] | <0.001 | 1.65 | [0.93; 2.91] | 0.087 |
| Chronic back problems | 1.26 | [0.76; 2.09] | 0.367 | 1.98 | [1.68; 2.33] | <0.001 | 1.69 | [1.14; 2.49] | 0.009 |
| Depression | 2.99 | [1.75; 5.09] | <0.001 | 1.32 | [1.08; 1.62] | 0.007 | 2.65 | [1.62; 4.35] | <0.001 |
| Lung disease | 1.65 | [0.67; 4.05] | 0.273 | 1.25 | [0.71; 2.19] | 0.442 | 1.04 | [0.40; 2.68] | 0.940 |
| Cancer | 0.94 | [0.36; 2.44] | 0.893 | 1.27 | [0.94; 1.71] | 0.119 | 2.24 | [0.79; 6.34] | 0.128 |
| Chronic renal insufficiency | 0.13 | [0.02; 0.95] | 0.044 | 0.97 | [0.61; 1.54] | 0.886 | 0.32 | [0.11; 0.92] | 0.035 |
|  |  |  |  |  |  |  |  |  |  |
| PROXIMAL MODEL (*n* = 58,157) ^c^ |  |  |  |  |  |  |  |  |  |
| *Self-perceived health status* |  |  |  |  |  |  |  |  |  |
| Good / very good | 1 |  |  | 1 |  |  | 1 |  |  |
| Regular | 0.62 | [0.36; 1.07] | 0.086 | 1.35 | [1.17; 1.56] | <0.001 | 0.81 | [0.50; 1.34] | 0.415 |
| Bad / very bad | 1.02 | [0.30; 3.51] | 0.974 | 1.52 | [1.17; 1.98] | 0.002 | 0.49 | [0.21; 1.14] | 0.097 |
|  |  |  |  |  |  |  |  |  |  |
| Abbreviations: “N” or “n”: sample size; “OR”: odds ratio; “95% CI”: 95% confidence intervals.  ^a^ Model run with the variables of accessibility, sex, age, ethnicity, per capita household income, and educational attainment.  ^b^ Adjusted by accessibility, sex, age, ethnicity, per capita household income, and educational attainment.  ^c^ Adjusted by accessibility, sex, age, ethnicity, per capita household income, educational attainment, and chronic diseases. | | | | | | | | | |
